# A mobile and web-based clinical decision support and monitoring system for diabetes mellitus patients in primary care: a study protocol for a randomized controlled trial

**DOI:** 10.1186/s12911-017-0558-6

**Published:** 2017-11-29

**Authors:** Özge Kart, Vildan Mevsim, Alp Kut, İsmail Yürek, Ayşe Özge Altın, Oğuz Yılmaz

**Affiliations:** 1 Department of Computer Engineering, Dokuz Eylül University, İzmir, Turkey; 2 Department of Family Medicine, Dokuz Eylül University, İzmir, Turkey

**Keywords:** Diabetes, Clinical decision support, E-health, M-health

## Abstract

**Background:**

Physicians’ guideline use rates for diagnosis, treatment and monitoring of diabetes mellitus (DM) is very low. Time constraints, patient overpopulation, and complex guidelines require alternative solutions for real time patient monitoring. Rapidly evolving e-health technology combined with clinical decision support and monitoring systems (CDSMS) provides an effective solution to these problems. The purpose of the study is to develop a user-friendly, comprehensive, fully integrated web and mobile-based Clinical Decision Support and Monitoring System (CDSMS) for the screening, diagnosis, treatment, and monitoring of DM diseases which is used by physicians and patients in primary care and to determine the effectiveness of the system.

**Methods:**

The CDSMS will be based on evidence-based guidelines for DM disease. A web and mobile-based application will be developed in which the physician will remotely monitor patient data through mobile applications in real time.

The developed CDSMS will be tested in two stages. In the first stage, the usability, understandability, and adequacy of the application will be determined. Five primary care physicians will use the developed application for at least 16 DM patients. Necessary improvements will be made according to physician feedback. In the second phase, a parallel, single-blind, randomized controlled trial will be implemented. DM diagnosed patients will be recruited for the CDSMS trial by their primary care physicians. Ten physicians and their 439 patients will be involved in the study. Eligible participants will be assigned to intervention and control groups with simple randomization. The significance level will be accepted as *p* < 0.05. In the intervention group, the system will make recommendations on patient monitoring, diagnosis, and treatment. These recommendations will be implemented at the physician’s discretion. Patients in the control group will be treated by physicians according to current DM treatment standards. Patients in both groups will be monitored for 6 months. Patient data will be compared between 0th and 6th month of the study. . Clinical and laboratory outcomes will be assessed in person while others will be self-assessed online.

**Discussion:**

The developed system will be the first of its kind to utilize evidence based guidelines to provide health services to DM patients.

**Trial registration:**

ClinicalTrials.gov NCT02917226. 28 September 2016.

## Background

Diabetes mellitus (DM) is among the leading causes of morbidity and mortality worldwide. A study from the National Burden of Disease reports that cardiovascular diseases and diabetes rank first and ninth among causes of death, with mortality rates of 47.7% and 2.2%, respectively [1]. In 2009, The World Health Organization reported that the top three risk factors for mortality are, in order, high blood pressure, smoking, and high blood glucose [2]. Diabetes has a high prevalence and continues to increase worldwide over time.

According to a study by TURDEP II, 13.7% of the Turkish population has diabetes [3]. HbA1c is accepted as a marker for glycemic control criterion. Ratio of diabetic patients whose blood glucose is regulated is very low [3]. According to a study, the rate of diabetic patients whose HbA1c value is <7% were found only 30.1% [4].

Diabetes is one of the highest cardiovascular disease risk factors. If the disease is taken under control, morbidity and mortality rates of society reduce. Despite the high rate of DM prevalence, diagnosis of the disease is still around 13.7%. The diagnosis rate should be increased first to reduce morbidity and mortality caused by the DM disease. DM prevalence studies in Turkey have shown that halves rule is valid for the awareness, treatment and control rates of the disease [3].

Today, the purpose of treatment of chronic illness is not to heal the patient, but to enhance the adherence of the individual to the treatment program and to promote the life quality by cooperation. For this reason, the importance of protecting, maintaining and developing health has been emphasized over the treatment of disease, thus nurturing the concept of “self-care” [5].

Diabetes is a chronic health problem. Diabetes patients often face challenges throughout treatment, including emotional instability and difficulty adapting to lifestyle changes. Except for the natural distress caused by symptoms, complications and treatments, the patient’s future worries affect his cognitive and emotional functions and social life [6]. Self-efficacy beliefs play an important role in individuals with health problems like diabetes that require complex treatment and care, taking steps to make lifestyle changes and learning new skills to cope with the disease process. It is expected that diabetics should have sufficient self-efficacy to cope effectively with complex diabetes care and treatment. Diabetics‘self-care behaviors can be improved by increasing their self-efficacy levels [7, 8].

Guidelines used for DM management are prepared on the basis of evidence based studies conducted by experts both at the national and international level.

In DM management guidelines, the standards for diagnosing are specified and the requirements for differential diagnosis are systematized. [9]. Then the disease is classified according to the patient’s history, physical examination and laboratory data.

The guidelines suggest which patient risk factors should be evaluated and which tests should be completed. After diagnosis and differential diagnosis, treatment alternatives are suggested to the physician based on the guidelines set forth by the evidence-based studies. These guidelines include systematical treatment and follow-up process according to the diagnosis and prognosis. [9, 10].

Physicians’ guideline use rate in Turkey, as well as internationally, is very low. A study conducted in Turkey showed that diabetic patients are not treated according to current guidelines [11].

Periodic health care is a monitoring program formatted by risk factors, through screening, examination and testing of healthy people, counseling and health education. The program is evidence based, effective, feasible and acceptable. In this evidence based program, screening, case detection, immunization, chemoprophylaxis and counseling are defined clearly according to people’s age, sex and risk factors. Institutions that provide primary care carry out periodic health care services. Established guidelines require healthy adults to be periodically screened for health risk assessments. For example, the HbA1C test must be applied once every three to five years to patients with a high risk score, and once a year to those with a very high score [12].

Primary care providers play a vital role in the diagnosis, treatment and control of DM disease. The World Health Organization states that primary health care has a key role in reducing the morbidity and mortality of chronic diseases. Strengthening primary care is on the agenda of all countries for ensuring the control of chronic diseases. Therefore, adapting the physician’s approach to current DM management guidelines and developing a standard approach will provide significant gains in diabetes control. Control of diabetes will increase the length and quality of patient life. This will allow patients to apply less to primary health care providers. Thus, the time that physicians will allocate per patient will increase.

There are several computerized clinical decision support systems (CDSS) developed for management of chronical diseases [13]. There are a variety of uses of CDSS in primary care such as depression, hypertension and medication reviews [14–16]. In addition, various rule based and artificial intelligence based decision support systems have been developed for diabetes diagnosis [17, 18].

Mobile technologies are becoming important for providing individual-level support to health care users and promising platform for health interventions. In recent years, researchers have been using mobile phones as tools for encouraging physical activity and healthy diets [19], for symptom monitoring in asthma [20] and heart disease [21], reminding patients about upcoming appointments, supporting smoking cessation [22], and for a range of other health problems [23]. In addition, various kinds of m-health applications were developed for other purposes such as enabling users to log and chart data about their diet [24, 25], exercise [26, 27], blood glucose levels [28], and other health-related behaviors and measures [29–32]. Examples included patient terminals for tele-monitoring of conditions such as hypertension [33] and chronic heart failure [34] and applications that receive data from pedometers, blood pressure monitors and other devices [35]. On the other hand, some researchers [36] developed a diabetes support system using mobile phones, in which the user inputs information regarding his/her daily measures rather than using sensors.

### Study aim

The purpose of the study is to develop a user-friendly, comprehensive, fully integrated web and mobile-based Clinical Decision Support and Monitoring System (CDSMS) for the screening, diagnosis, treatment, and monitoring of DM diseases which is used by physicians and patients in primary care and to determine the effectiveness of the system.

The clinical decision support system is developed based on the rules obtained from evidence-based guidelines [10]. The web based system provides screening, diagnosis, and treatment suggestions to primary care physicians. For (tele) monitoring system, a mobile health application will be developed to collect patients’ measures both manually and via sensors.

## Methods

### Cdsms

The clinical decision support rule sets are built by three family medicine research academicians and a consultant cardiologist using evidence-based guidelines. Then, three computer engineering researchers develop a web and mobile based software using these protocols. This software will be used by physicians and patients. The software has been developed and it is currently in the testing process.

### Design and settings

The study design is a parallel single blind randomized controlled trial**.** The research will be conducted in Dokuz Eylül University (DEU) Family Medicine Outpatient Clinic and selected family medicine units in İzmir. DEU Hospital is a university hospital in Izmir, the third largest city in Turkey. The family medicine outpatient clinic provides primary healthcare services to outpatients in the DEU department of family medicine.

The family medicine units which provide primary health care are located widely in Izmir. There are 972 family medicine units in Izmir metropolis. They serve under Turkish Ministry of Health. In Turkey, electronic health record systems are used for the health care services of the family medicine units. These systems mainly records preventive health care services, disease diagnoses and drug related data. Because there is not a governmental obligation, these systems have not any evidence-based sets.

In this study, validation of the developed CDSMS will be done in two stages. In first stage, the understandability and usability and adequacy of the system will be tested. Necessary adjustments will be made in the program in accordance with feedbacks taken from physicians and patients who use the application.

**Fig. 1:**
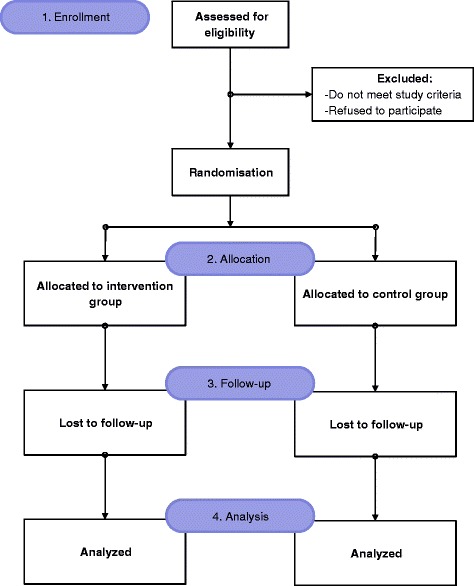
CONSORT Randomization Schema [37]

In the second stage, effectiveness of CDSMS will be evaluated by conducting a parallel single blind randomized controlled trial. Consort schema [37] of the RCT is in Fig. 1.

### Participants and sample size

The study population consists of patients applied to Dokuz Eylül University Family Medicine Clinics and family medicine units in İzmir. The CDSMS which is developed in this study will be validated in two stages. Different sample numbers and sampling methods will be used in each stage.

### Stage 1

In the first stage, understandability, usability and adequacy of CDSMS software will be tested. In this stage, two researchers from Department of Family Medicine and three physicians from other family medicine units will test CDSMS on at least 16 diabetes patients. Inclusion and exclusion criteria for stage 1 are listed below in Table 1. The CDSMS software test will be done on diabetes patients who apply for consultation. The physicians will perform patient interviews using the CDSMS. They will fill in feedback forms about program functionality and improvements needed. A Web Site Usability Test [38] and feedback form will be used to measure understandability, usability, and adequacy of CDSMS through user perception. The scale has 25 items. The answers to scale items are gathered with Likert type quintile rating scale. ISO/IEC 9126–1 (2001) defines usability as the capacity of the software product to be included/understood, learned, used and attractive to the user, when it is used under specified conditions. It characterizes the four attributes of usability as understandability, learnability, operability, and attractiveness of the software product. Understandability assesses whether new users can understand whether the software is suitable and how it can be used for particular tasks. Learnability assesses how long it takes users to learn to use particular functions, and the effectiveness of help systems and documentation. Operability assesses whether users can operate and control the software. Attractiveness metrics assess the appearance of the software, and will be influenced by factors such as screen design and color [39]. The feedback form will consist of open-ended questions and will be used to get feedback and suggestions from the users.

**Table 1:** Inclusion and exclusion criteria

Inclusion Criteria	Exclusion Criteria
• Over age 40 • Volunteer • Computer and Internet literacy • DM diagnosed or having at least one of diagnostic criteria. These criteria; • HbA1c ≥ 6.5 • FPG ≥ 126 mg / dl • 2-h postprandial glucose ≥200 mg / dl • Any time postprandial glucose ≥200 mg / dl • Diabetes symptoms	• Having communication problems • Type 1 diabetes • MODY type DM • Psychotic disorders • Dementia

### Stage 2

In the second stage, the health data of 3000 participants will be evaluated to determine the effectiveness of CDSMS in screening. Participants who use medicine for DM treatment will be excluded from data analysis when measuring the effectiveness of the system in screening.

Patients who applied to primary health care services for any reason and screened within the scope of periodic health care for diabetes, having at least one measurement of FPG (Fasting Plasma Glucose), HbA1C or PPG (Postprandial Plasma Glucose) in last six months will be participants of stage 2. Participants determined in second phase will be evaluated in terms of inclusion and exclusion criteria. Eligible and volunteer participants will take part in the trial. Inclusion and exclusion criteria for randomized controlled trial are listed below in Table 1.

Patients who were diagnosed with DM among 3000 participants (new diagnosis, former diagnosis, medicine users, non-medicine users) will form the population of the randomized controlled trial. The sample size evaluated by significance level *p* = 0.05, 80% power, 25% prevalence (controlled diabetes) and odds ratio 2 will be included to experimental study. Because in RCT studies lost to follow up is approximately 15.0%, this amount is taken into account for determining the sample size. The sample contains 439 patients with 110 interventions and 329 control groups. The ratio of intervention to non-intervention (controls) is 1:3. About 3000 people over age 40 apply to the 10 primary health care units (In the city of Izmir in Turkey) in a month. For reaching expected number of samples, at least %14 of patients are thought to be eligible for inclusion criteria. (assuming DM prevalence approximately 14%).

Diabetes symptoms aforementioned in Table 1 are classical symptoms of polyuria, polydipsia, polyphagia or loss of appetite, weakness, fatigue, dry mouth, nocturia. Less common symptoms are blurred vision, unexplained weight loss, persistent infections, recurrent yeast infections and itching [10].

#### Enrollment

Patients with age above 40 who applied to family medicine units for any reason and screened within the scope of periodic health care for diabetes are eligible for the study. Guidelines determine that 40 is the age to start diabetes screening for people without having any risk factors [9, 10].

Around 3000 people over the age of 40 apply to the 10 family medicine units in Izmir, Turkey each month. At least 14% of these people (DM prevalence is accepted about 14%) are expected to be eligible for inclusion criteria. 3000 individuals will be evaluated in terms of inclusion and exclusion criteria. Thus, the expected sample size will be reached.

Excluded and refused to participate: Selected participants after being assessed for eligibility will be asked about voluntariness for intervention. They will be taken informed consent. Then the participants will be taken to randomization.

#### Allocation

Eligible participants will be assigned to intervention and control groups with simple randomization using a computerized random number generator for assignment into two groups. 110 patients will be allocated to the intervention group and 329 patients to the control group. Three physicians will treat and monitor intervention group using the CDSMS. In the control group, seven physicians will treat and monitor without using CDSMS.

Researchers will determine the random allocation sequence and make lists of sequence. These lists will be given to the nurses of the family medicine center. The nurses will assess the patient according to eligibility criteria and allocate eligible patients to either intervention or control groups depending on the lists.

#### Follow-up

Patients in both groups will be followed up for 6 months. Data from 0th month and 6th month will be compared.

#### Analysis

Various comparative analyzes will be performed using data obtained from both groups and excluded from follow-up.

### Procedure

#### Stage 1

In the first stage, understandability, usability and adequacy of CDSMS software will be tested. 5 physicians will test CDSMS on at least 16 diabetes patients. The system will be revised according to the comments received on the Web Site Usability Test [38] and feedback form.

#### Stage 2

At this stage, a randomized controlled trial will be performed. Firstly, the physicians who manage the treatment of the patients will register online through the web based portal for free. The researchers will train these physicians about the use of portal.

Patients who are referred to select family medicine centers for any reason will receive diabetes screening using CDSMS. The patients who were previously diagnosed as diabetics will be recruited for trial by the physicians. The recruited patients will register to the CDSMS via mobile phone or web page with the accounts provided by their physicians, free of charge.

**Fig. 2:**
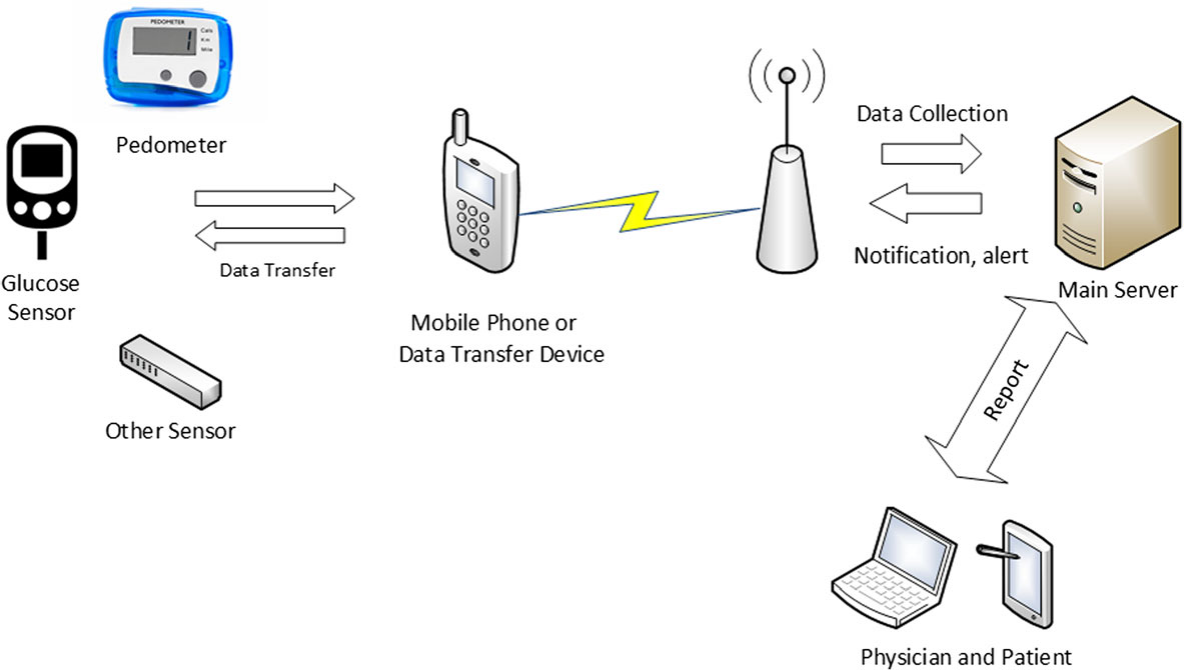
Tele-monitoring system

The proposed clinical decision support system is a recommended tool for primary care physicians to make clinical decisions on screening, diagnosis, treatment and monitoring of DM patients. Physicians input the data of patients having FPG, HbA1c or PPG test results in last six months. CDSS will make a statement on diagnosis of patient and/or stage. The system will also suggest a treatment plan for the patient. The physician will evaluate the system’s recommendations and decide on a treatment for the patient and input his/her treatment decision. CDSS will suggest a monitoring plan to the physician. The physician will evaluate the proposals and input the monitoring plan that he/she has decided for the patient into the system. The follow-up visits will happen with the physician. There are two options for carrying out the monitoring process:

The first is installing the developed mobile application. Patients will install the application on their mobile phone and complete the online registration process. They will be trained about how to access and use the program and how to input their data into the daily program on the application. They will be given a brochure which briefly explains the program. Then, usual patient examination process will be completed. Patients will input their measurement data (e.g. FPG, diet, exercise, weight) regularly or these data will be transferred automatically from sensors to the system via communication technologies such as Bluetooth, Wi-Fi etc. (Figure 2). Through mobile application, patients can receive individualized therapy as well as alerts and notifications such as the amount and frequency of use of medicines on their monitoring process.

The second is using a transfer device developed for this study. The transfer device will be developed for especially elderly patients who are not able to use smart phones. It is specialized for data collection and transferring. This device will read data automatically from sensors which have communication ability (e.g. Bluetooth) or measured values will be entered manually via large buttons for the ease of use for the elderly.

These options will allow patient data to be transferred to the system periodically from either a mobile application or a transfer device. Regularly collected data will be used for physician’s evaluation. Notifications will be sent via web and mobile application to the physician and/or patient when deviations from the normal state occur. Notification messages will be in the form of reminders, including reminders to take medication, follow the diet, and complete exercise. It will also notify the patient when blood sugar is high, and will notify both the patient and the physician if the patient is experiencing a risky situation such as hyperglycemia or hypoglycemia. The CDSMS will generate the notifications according to the patient’s input, processing the algorithms defined in the system according to the evidence based guidelines. In accordance with the patient data in the system, notices and short reports will be created for physician and patient in certain periods of time.

For patient security, personal information will be kept encrypted. In addition, technologies such as https, VPN will be used during the secure access to the system. Access rights to the system will be determined by user role definitions.

When testing CDSMS, the system will make a suggestion to the physician. The physician will make his/her own decision on the patient considering the system’s suggestion and his/her clinical experience. Both the suggestion of CDSMS and physician’s own decision will be saved in system and compared later. Physicians will take suggestion of CDSMS but implement their own decision for each case. The application is designed in this specific way in order to avoid any harm on patients.

No side effects are expected depending on the intervention. However, the intervention is discontinued due to hospitalization in cases of diabetes or other illnesses. In addition, the intervention is ended due to participant request or death.

There is no plan for terminating the trial. Because there is no specific risk or harm expected from the intervention. If a disease other than diabetes occurs during the trial, the routine health care necessary for the treatment of the disease is applied.

### Measures

Primary outcomes of RCT include HbA1c, Fasting Blood Glucose and Postprandial Blood Glucose levels. Secondary outcomes include body mass index, diabetes-related emotional burden and interpersonal distress sub-scales, self-efficacy for diabetes self-care, blood glucose monitoring, physical activity, nutrition, medication-taking, diabetes self-care: blood glucose self-monitoring, medication-taking, smoking and alcohol using and physical activity, depression, anxiety, stress levels. Clinical and laboratory outcomes will be face-to-face assessed, others will be online self-assessed. The effectiveness of the system will be evaluated by using primary and secondary outcomes.

Table 2 lists all the outcomes and measures used in this study. Fasting blood glucose, HbA1c and other tests according to patient history and physical examination will be determined and implemented for each patient taken into intervention and control groups. Body mass index of patients will be calculated. On baseline all scales will be implemented online such as Diabetes Distress Scale [40], Diabetes Self-Efficacy Scale [41], Diabetes Self-Care Activities Survey [42], Morisky Medication Adherence Scale [43], The Patient Assessment of Chronic Illness Care-Patient Form [44, 45]. Monthly average of FPG and PPG values of patients regularly taken from the tele-monitoring system will be evaluated. Glycosylated hemoglobin (HbA1c) test will be done quarterly.

**Table 2:** Measures

Measurement area	Outcomes assessed	Measure/s
Demographics	Age, gender, nationality, country of birth, relationship status, employment status, health insurance	Short answer and multiple choice items
Laboratory	Glycosylated hemoglobin A1c (HbA1c) level Fasting And Postprandial Blood Glucose	Venous blood sample
Clinical	Height, Weight, Diabetes Symptoms, Risk Factors	Patient history and physical examination
Emotional/ Psychological	Depression, anxiety, stress levels Diabetes-related emotional burden and interpersonal distress sub-scales Self-efficacy for diabetes self-care: blood glucose monitoring, physical activity, nutrition, medication-taking	Diabetes Distress Scale [40] Diabetes Self-Efficacy Scale [41]
Behavioral	Physical activity Diabetes self-care – blood glucose self-monitoring, medication-taking, smoking and alcohol using and physical activity	Diabetes Self-Care Activities Survey [42] Morisky Medication Adherence Scale [43]
Patient therapy evaluations	Patients’ satisfaction with therapy	The Patient Assessment of Chronic Illness Care-Patient Form [44, 45]
User program evaluations	Users’ program usage, perceived utility and acceptability, ease of use, user interface, and satisfaction with program	Web Site Usability Scale [38] Feedback form

Participants in the intervention group will be given screening, diagnosis, treatment and monitoring services by the physicians using CDSMS for six months. These patients will be monitored in the framework of the protocols. The control group patients will receive services from physicians as they have done before without further intervention. All tests and scales implemented on screening and baseline will be repeated at the end of six months. By comparing the two groups, the effectiveness of the study will be measured. The measures and scales used at screening (enrollment), baseline, monthly, quarterly and at the end of intervention are summarized in Table 3.

**Table 3:**
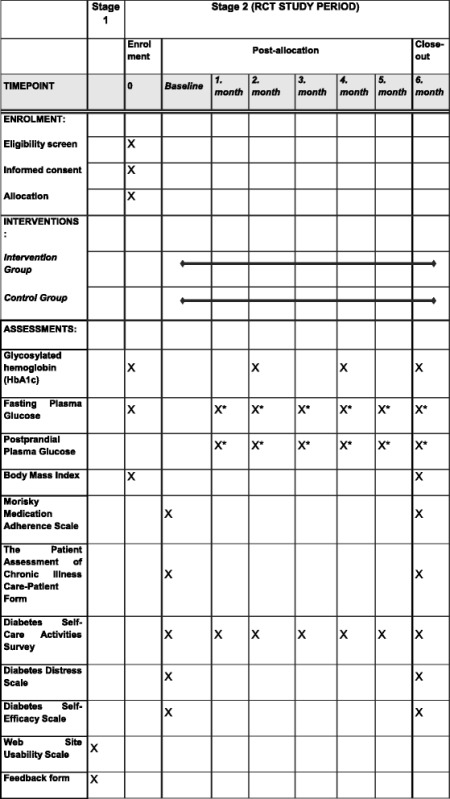
Schedule of enrolment, interventions, and assessments

*monthly average values of daily measurements collected by mobile application

### Statistical analysis

Statistical analysis will be performed using SPSS software. As stated in the method section, the system will be tested in two stages.

### Stage 1

For the first stage, the comments in the feedback forms will be used for revisions of program. Descriptive analyzes will be done for web site usability test results.

### Stage 2

At the end of the intervention, independent samples t-tests will be performed to measure the difference between the intervention and control groups. On both groups, one-sample t-test will be performed to measure the difference between baseline and post intervention in the same group.

Each of the dependent measures of primary outcomes will be analyzed using a generalized linear mixed model involving a single between Group factor (Treatment vs. Control), a single within Period factor (enrollment vs. end of 6 months), and interaction between Group and Period factor. Categorical data of both control and intervention groups will be analyzed together using logistic regression. The rates of controlled and uncontrolled diabetes patients will be determined.

An intention-to-treat analysis will be performed. The analysis will include all individuals’ data. In the analysis, patients will be compared regardless of patient compliance, crossover to other treatments, or withdrawal from the study.

## Discussion

This study describes the first comprehensive clinical decision support system intended for diabetes screening, diagnosis, treatment and monitoring in Turkey. Moreover, this clinical decision support and monitoring system using evidence-based guidelines will pave the path for other medical specialties providing health services to diabetes patients. In addition, remote monitoring of patient data and alert mechanisms are also implemented in this study. Patient adherence to treatment in medication and lifestyle changes will be provided.

Using CDSMS based on current diabetes guidelines and telemonitoring systems will provide patients a means to receive effective treatment and regular monitoring. This will reduce possible complications, hospitalization, morbidity, and mortality caused by diabetes while also reducing the cost of treatment.

Electronically monitoring period screenings will provide early diagnosis and protection from potential diseases. This will enable patients to live longer and healthier lives, reduce treatment costs, and prevent the labor loss.

In CDSMS, patients actively participate in their treatment by regularly inputting measurement values and receiving motivational and reminder messages. Constant communication will increase the patient’s self-efficacy, thereby reducing emotional stress and increasing the patient quality of life.

Information technologies are integrated to patient care and monitoring in primary health care services with the proposed CDSMS based on predictive modeling. This new comprehensive and integrated approach will contribute to rapidly evolving e-health technology in the world using national information and technology savings. In addition, patient data will be collected when using rule based CDSMS. As a future study, an intelligent clinical decision support system will be developed using machine learning and artificial intelligence technologies on collected data.

## Abbreviations

CDSMS: Clinical decision support and monitoring system
DM: Diabetes Mellitus
RCT: Randomized controlled trial
